# Geographic accessibility to childhood tuberculosis care in Pakistan

**DOI:** 10.1080/16549716.2022.2095782

**Published:** 2022-07-18

**Authors:** Aashifa Yaqoob, Muhammad Rizwan Alvi, Razia Fatima, Hina Najmi, Zia Samad, Nadia Nisar, Anwar Ul Haq, Basharat Javed, Abdul Wali Khan, Sven Gudmund Hinderaker

**Affiliations:** a Research Unit, Common Management Unit [TB, HIV/AIDS & Malaria], Islamabad, Pakistan; b Department of Global Public Health and Primary Care, University of Bergen, Bergen, Norway; c Digital Security & Intelligence, Inbox Business Technologies, Islamabad, Pakistan; d Maternal Newborn and child Health, Health Services Academy, Islamabad, Pakistan; e M & E and Surveillance, Common Management Unit (TB, HIV/AIDS & Malaria), Islamabad, Pakistan; f International Health Regulations Strengthening project, Public Health England, Islamabad, Pakistan; g Directorate of Central Health, Ministry of National Health Services Regulation & Coordination, Government of Pakistan, Islamabad, Pakistan; h National TB Control Program, Common Management Unit (TB, HIV/AIDS & Malaria), Islamabad, Pakistan

**Keywords:** Tuberculosis, children, access, secondary, tertiary care level, public sector, distance, health facilities, settlements

## Abstract

**Background:**

Tuberculosis (TB) in children is difficult to detect and often needs specialists to diagnose; the health system is supposed to refer to higher level of health care when diagnosis is not settled in a sick child. In Pakistan, the primary health care level can usually not diagnose childhood TB and will refer to a paediatricians working at a secondary or tertiary care hospital. We aimed to determine the health services access to child TB services in Pakistan.

**Objective:**

We aimed to determine the geographical access to child TB services in Pakistan.

**Method:**

We used geospatial analysis to calculate the distance from the nearest public health facility to settlements, using qGIS, as well as population living within the World Health Organization’s (WHO) recommended 5-km distance.

**Result:**

At primary health care level, 14.1% of facilities report child TB cases to national tuberculosis program and 74% of the population had geographical access to general primary health care within 5-km radius. To secondary- and tertiary-level health care, 33.5% of the population had geographical access within 5-km radius. The average distance from a facility for diagnosis of childhood TB was 26.3 km from all settlement to the nearest child TB sites. The population of one province (Balochistan) had longer distances to health care services.

**Conclusion:**

With fairly good coverage of primary health care but lower coverage of specialist care for childhood TB, the health system depends heavily on a good referral system from the communities.

## Background

Every day, more than 650 children are estimated to die from tuberculosis (TB); 96% of them do not get TB treatment [1]. Children with TB are often not diagnosed and reported due to multiple factors like limited capacity of health care providers, unavailability of child health services, lack of trained clinician, non-specific symptoms overlapping with other common childhood diseases, complex diagnostic algorithms, lack of a sensitive point-of-care test, and limited contact-tracing activities [2,3].

Pakistan ranks sixth among countries with the largest contributions to the global shortfall in TB notifications in 2020 and reported incidence of 259 per 100,000 new TB cases annually with 48% of them getting treatment [4]. Of the total load of TB cases, children accounts 11%, with 9.9 million incidence rate [4]. Majority of the population in Pakistan has geographical access to primary health care (PHC), and a child with presumptive TB is recommended referral to a secondary or tertiary care hospital with diagnostic services and paediatricians [5].

Geographical distance to health care has been linked to treatment delay and poor adherence to TB management plans [6–9]. One bottleneck in the management of childhood TB in Pakistan is the lack of a systematic mechanism to refer children with presumptive TB from PHC facilities to the facilities where childhood TB diagnostic services are available. Therefore, understanding the link between geographic distance and coverage of childhood TB services may be useful to make evidence-based health policies that could reduce barriers to childhood TB care and improve their outcomes across Pakistan. A number of studies have explored access to health facilities in Pakistan with different perspectives [10–14]. Empirical quantitative information on health care distribution, geographical accessibility, and equity of general and child TB care remains generally scare. Therefore, this study aimed to measure the distance from community centres to health facilities with childhood TB care in Pakistan by using spatial analytical techniques. Our specific objectives were as follows: 1) to measure the distance from community centres to PHC facilities and to childhood TB services and 2) to measure the population coverage within 5 km for PHC and for childhood TB services.

## Methods

### Study design

This was an ecological study design based on retrospective record of different source to determine the health services geographical access to general and child TB services in Pakistan using secondary data.

### General setting

Pakistan is the sixth largest country of the world having 207 million population with an annual growth rate of 2.4% [15]. Out of this, 37% live in urban areas, while a significant portion (63%) resides in rural areas. The country is administratively divided into the Islamabad Capital Territory (ICT); four provinces: Balochistan (with 33 districts), Khyber–Pakhtunkhwa (KP, with 34 districts), Punjab (36 districts), and Sindh (29 districts) and two regions: Gilgit-Baltistan (GB with 10 districts) and Azad Jammu and Kashmir (AJK with 10 districts)], and the Federally Administered Tribal Areas (FATA) are merged with KP from 31 May 2018. The four provinces, capital territory, and two autonomous territories of Pakistan are subdivided into 37 administrative ‘divisions’, which are further subdivided into districts, tehsils, and finally union councils. The divisions do not include the ICT or the FATA, which were counted at the same level as provinces.

Pakistan has a mixed health system, which includes government (public) infrastructure, parastatal health institutions, the private sector, civil society, and philanthropic contributors. Public health care is delivered in the provinces mainly through a chain of primary-, secondary-, and tertiary-level health facilities. PHC facilities include civil dispensaries, basic health units (BHU), rural health centres, maternal and child health centres, urban health units, and urban health centres. The secondary-level health care facilities comprise taluka (tehsil-sub-district level) hospitals and district hospitals. Tertiary-level health care is provided through teaching and specialized hospitals. The private health sector is large and unregulated, comprising qualified and unqualified service providers; it is estimated that 75% of general curative services are from private sector [16].

### Specific setting

The Pakistan National TB Control Program (NTP), with the support of provincial TB programs (PTPs), is responsible for TB care and control activities that are integrated into PHC at district level. This integration has made it possible to plan and carry out TB control in a district without the addition of TB-specific care delivery staff. The district TB team is primarily responsible for advocating, planning, financing, implementing, and monitoring TB care services in their respective districts. In Pakistan, facilities where children with TB can be diagnosed and managed (Child TB sites) are secondary and tertiary care facilities.

### Data sources and collection

**Figure 1.**
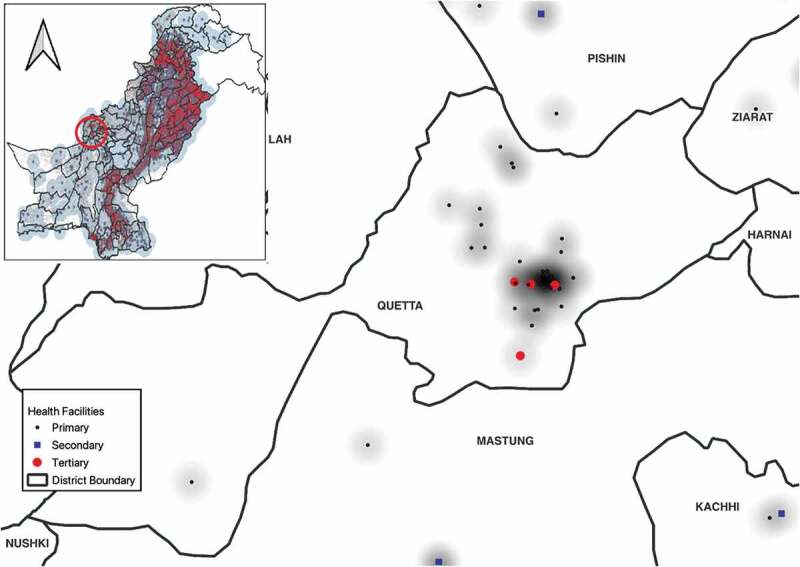
Distribution of primary, secondary, and tertiary health facilities in Pakistan.

- The list of public health facilities in Pakistan was obtained from the District Health Information System (DHIS) and matched with number of health facilities reported by provincial health department. Of these, 1283 health facilities engaged with NTP (Table 1). Geographical coordinates of all public health facilities were derived from publicly available data source The Humanitarian Data Exchange (https://data.humdata.org/organization/alhasan-systems-private-limited). This database was matched/cross-verified with list of public health facilities reported in DHIS, and missed health facilities were mapped manually using Google Maps. Distribution of health facilities in Pakistan is shown in Figure 1.
- Population density mapping: Since there is no official source available accounting for the population in the cities and sub-districts level, we used the grid population data from LandScan. This provides gridded population estimates range in size from 30 × 30 mm to 1 × 1 km; it is freely available for researchers (https://landscan.ornl.gov/). These estimates are generated through spatial modelling and image analysis with inputs from census data, high-resolution imagery, land cover, and other spatial data such as various boundaries, coastlines, elevations, and slopes [17].
- Spatial geographical accessibility analysis: Pakistan settlement data were obtained from publicly available dataset on The Humanitarian Data Exchange website (https://data.humdata.org/dataset/pakistan-settlement). The dataset contains the settlements/locations across Pakistan with Province-, District-, and Tehsil-level details; there are approximately 261,217 geographical coordinates of settlements covering four provinces and ICT in Pakistan. GB and AJK settlements are not covered in this dataset. The source of the dataset is World Gazetteer – National Geospatial-Intelligence Agency. Settlement defines as a colony, a town, a village, some small area in city, or any small community of people.

**Table 1.** Number of public health facilities and their engagement with NTP in Pakistan, by province or region, 2021.

				Primary Health Care Level								
Province/region	Public Health Facilities			Basic Health Units (BHUs)			Rural Health Centres (RHCs)			Secondary and Tertiary Care Level		
	Total	Engaged with NTP		Total	Engaged with NTP		Total	Engaged with NTP		Total	Engaged with NTP	
		n	(%)		n	(%)						
Punjab	3062	516	(17.0)	2500	6	(0.24)	358	332	(92.7)	204	178	(87.3)
Sindh	1010	330	(32.7)	710	48	(6.7)	204	186	(91.2)	96	96	(100.0)
KPK	976	222	(22.7)	738	10	(1.3)	111	96	(86.5)	127	116	(91.3)
Balochistan	839	109	(13.0)	688	39	(5.7)	106	26	(24.5)	45	44	(97.8)
AJK	297	59	(19.8)	227	12	(5.3)	46	26	(56.5)	24	21	(87.5)
GB	59	40	(67.8)	13	6	(46.2)	14	14	(100)	32	20	(62.5)
Islamabad	23	10	(43.4)	16	3	(18.7)	3	3	(100)	4	4	(100.0)
Total	6266	1286	(20.5)	4892	124	(2.5)	842	683	(81.1)	532	479	(90.0)

### Outcome measurement

The WHO defines health services geographical access as per cent of population living within 5 km of a health facility and recommends everyone should have geographical access to a health facility within a 5-km radius [18]. The main primary outcome was the population living within 5-km radius from a health facility engaged with NTP (all vs child TB sites). Secondary outcome was summarising the distance from settlement centres to health facility engaged with NTP (all vs child TB sites).

### Analysis

**Figure 2.**
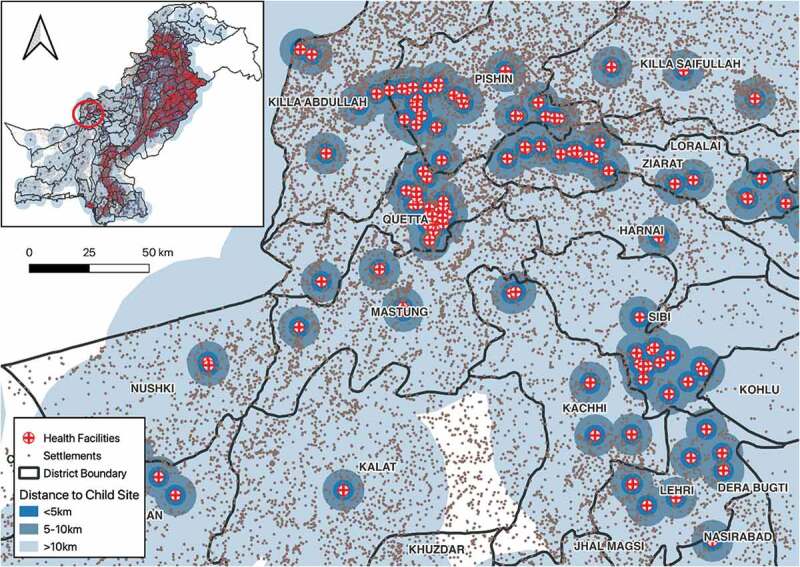
A geographical accessibility catchment zone (5-km buffer) of all health facilities in Pakistan.

**Figure 3.**
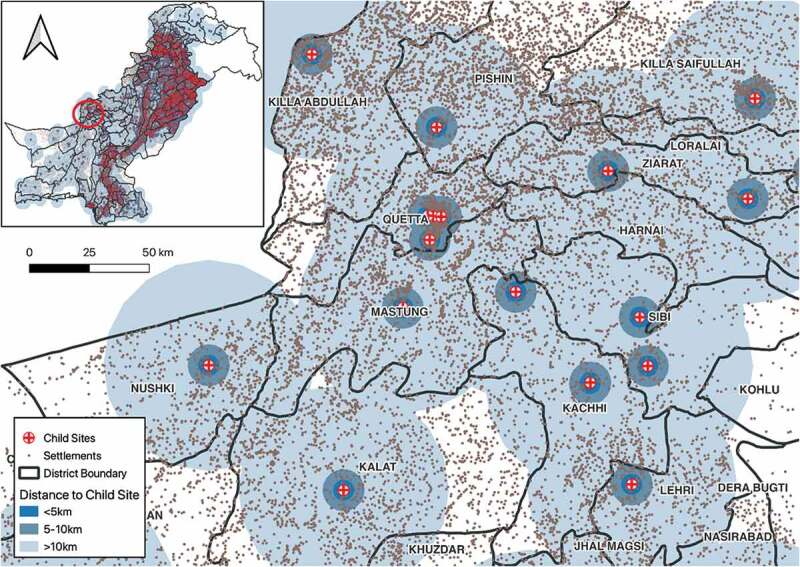
Distribution and geographical accessibility catchment zone (5 km buffer) of child sites (secondary and tertiary facilities) in Pakistan.

To determine the total population living within 5 km of a health facility engaged with NTP [19], 5 km dissolved buffer from the health facilities shapefile was generated using open source GIS software (qGIS) to create a geographical accessibility catchment zone (Figure 2 and 3). The geographical accessibility catchment zone was overlaid with the district shapefile using intersection tool in qGIS. The output of this operation was then geographically intersected with the population grid map (Landscan) using the Spatial Join and Summary Analyst Tool in qGIS. The total population in each district within 5 km of health facilities was extracted. The percentage of the total population that fell in the geographical access area was calculated and a choropleth map was created from the results. We repeated the same analysis with different subsets of health facilities (health facilities engaged with NTP that were child TB sites [secondary and tertiary health care facilities]).

Proximity information (median and interquartile range [IQR] distance) between settlements/communities and the nearest health facility engaged with NTP was calculated using the nearest neighbour analysis tool in qGIS which takes number of nearest settlements as input parameter and return mean max and summary of all nearest point to health facilities. We repeated the analysis, calculating distance between settlements/communities and health facilities engaged with NTP that were child TB sites.

## Results

Table 1 shows the number of public health facilities in Pakistan and those reporting and engaged with the NTP, by region and type of facility. Out of all public health facilities, 1286 (20.5%) were engaged with NTP. In primary care, NTP was primarily involved at rural health centres, whereas BHU level was very limited, except in the capital areas of Islamabad and the less populated GB province. At secondary and tertiary care level, NTP engagement was at 90%. PHC-level engagement with NTP was low except for Gilgit-Baltistan.

**Figure 4.**
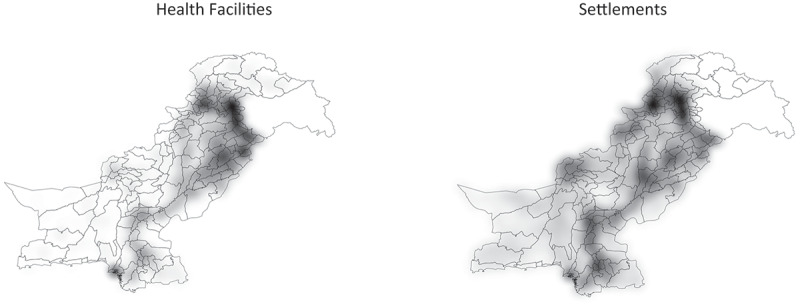
Heat map: Distribution of Health Facilities and Settlements in Pakistan.

Table 2 shows the proportion of community centres living closer than 5 km from a health facility. On average, in Pakistan, 74% lived closer than 5 km from health services. The province of Balochistan had longer distance to health facilities than all the other provinces; GB had the lowest proportion of its population living closer than 5 km from a health centre (26.5%), followed by Balochistan (51.8%). The overall average distance of all settlements to the nearest health facility in the study was estimated as 9.4 km. The province with the highest average distance to a health facility was Balochistan with 32.1 km, whereas for Punjab province, it was 5.9 km and for Islamabad, it was 3.6 km. A map of Pakistan showing health facilities and settlement is shown in Figure 4.

**Table 2.** Distance from geographical centre of communities to nearest public health facility, and proportion of communities closer than 5 km from a health facility, in Pakistan, 2020.

Province/region	Population	Population within 5 km*	(%)	Distance to nearest health facilities (km)**	
				Median	IQR
Punjab	107,389,208	84,799,769	(79.0)	5.9	3.8
Sindh	47,915,702	35,282,065	(73.6)	9.6	11.8
KPK	33,963,627	22,893,206	(67.4)	9.3	19.6
Baloschistan	9,586,794	4,967,299	(51.8)	32.1	30.2
AJK	4,831,880	3,045,826	(63.0)	-	-
GB	1,008,820	267,631	(26.5)	-	-
Islamabad	2,402,966	1,989,367	(82.8)	3.6	2.4
Pakistan	207,098,997	153,245,163	(74.0)	9.4	286.2

*Per cent of population living within 5 km of a health facility.

**Distance from geographical centre of community to health facilities. AJK and GB had no settlement data.

Table 3 shows the median distance to the closest health facilities giving childhood TB services in Pakistan, which are secondary and tertiary care hospitals. The total population within the 5-km buffer of child TB sites (secondary and tertiary) health facilities in Pakistan was estimated to be 69 million, representing 33.5% of the total population. The average distance of all settlement to the nearest child TB sites was estimated as 26.3 km. The median distance from communities to nearest facility to manage childhood TB was below 30 km in Punjab, Sindh, and KPK but almost 60 km in Balochistan.

**Table 3.** Distance from geographical community centre to the nearest health facility for childhood TB* in Pakistan, 2021.

Province/region	Population	Population within 5 km**	(%)	Distance of all communities*** to nearest child TB sites (km)	
				Median	IQR
Punjab	107,389,208	32,722,162.00	(30.5)	19.6	12.0
Sindh	47,915,702	21,671,105.00	(45.2)	25.8	23.1
KPK	33,963,627	9,363,649.00	(27.6)	23.1	28.5
Baloschistan	9,586,794	2,921,308.00	(30.5)	58.7	33.5
AJK	4,831,880	1,107,448.00	(22.9)	-	-
GB	1,008,820	260,320.00	(25.8)	-	-
Islamabad	2,402,966	1,239,151.00	(51.6)	8.6	7.8
Pakistan	207,098,997	69,285,143.00	(33.5)	26.3	449.2

*Child TB sites comprise secondary- and tertiary-level facilities where paediatrician and child TB diagnostic services are available.

**Per cent of population living within 5 km of a health facility.

***Distance from geographical centre of community to health facilities. AJK and GB had no settlement data.

## Discussions

This study found that PHC facility is available within 5 km for 74% of the general population. Management of children with TB is limited to secondary and tertiary care facilities, and only a third of the population lives within 5-km distance from these facilities. The median distance to a facility for the management of childhood TB is 26.3 km from settlements (community centre). According to guidelines [20], at PHC level, children with symptoms compatible with TB or severe unclear symptoms should be referred to higher level for further management.

In Pakistan, geographical access to diagnostic tools is often concentrated at and limited to secondary and tertiary care level. The current study estimated that 74% of the population had geographical access to general PHC but low geographical accessibility to secondary and tertiary care level. According to latest review, WHO mission report, the majority of physicians in Pakistan are not trained in TB case identification, follow-up, management, or the guidelines of the NTP [21]. Similar findings are evident in a study conducted in Ghana that describes the limited geographical access to secondary (61.4%) and tertiary care level (14.3%) [22]. Efforts should be made to reach all levels for identification/recognition of child TB cases, and childhood TB training should be incorporated within ongoing NTP training activities. There is also a need to strengthen referral networks between primary level facilities and those with diagnostic capacity of child TB cases at secondary and tertiary care level to improve child TB care geographical access [21].

This study estimated that only one third of the population living within 5km distance to secondary and tertiary health care facilities, but a majority of the population have geographical access to PHCs that provide only basic preventive and curative services, and importantly to refer cases they cannot diagnose or handle. According to the latest WHO Joint Mission report [21], the health staff of these PHC facilities are not trained and involved in the provision of any TB service including identification and referral of presumptive cases. In Sindh Province, however, it was observed that BHUs, whose staff were trained on the identification of presumed TB and linked to the relevant BMUs, were able to identify patients with TB signs and symptoms, use the register of presumed TB cases, and refer them to the closest BMUs. This experience strongly suggests that the involvement of PHC facilities is feasible. Most of the presumed TB patients who seek care in the BMUs visited during the WHO Joint Review Mission had not been detected by health-provider-initiated screening and referral from a PHC level; they were usually self-referred. This suggests that the process of TB case-finding is not taking place in the existing PHC network in Pakistan. In addition, the staff of the dispensaries and BHUs have little training in the management of the TB patients. As we see in Table 1, less than 10% of BMUs deal with NTP on regular basis, except special regions.

On average, people will have to travel 26 km to geographical access child TB services. The people living in Balochistan, AJK, and GB are more likely to have longer distances to geographical access child TB services, and this could lead to a significant burden in terms of time and money. An inventory study in Pakistan highlighted that 78% child TB cases were diagnosed by the non-NTP private providers, which may not be surprising when distance is long to public child TB care; childhood TB under-reporting was highest in these provinces [23]. We think telemedicine could be used remotely to link PHC to child TB sites for timely diagnoses and management of serious child TB cases and this could address some of the challenges posed by lack of physical health care infrastructure [24,25].

In general, in Pakistan, below the level of the rural health centre is not currently involved in TB services, representing a lost opportunity to bring TB services closer to the community and people affected by tuberculosis [21]. Many children are treated at home through the informal sector or by traditional healers. Studies consistently confirm that many sick children do not reach health facilities, and children from poorer families are less likely to obtain care [26]. The WHO recommend Integrated Management of Childhood Illnesses (IMCI) strategy [27] to be used in PHC sites, a community approach to TB prevention, case finding, and supportive care platform to ensure that all infants and children with TB receive high-quality care, and to ultimately eliminate TB deaths in children. The role of Lady Health Workers (LHWs) in referring individuals with presumptive TB from communities to qualified public providers has been well documented [28,29]. The LHWs who are usually linked to PHC facilities and community can play an important to connect community with PHC LHWs to improve referral of persons suspected to have TB from the community to primary health facilities, to support DOTS and report adverse reaction and for household contact tracing in community. Case studies from Malawi and Uganda also illustrated the successful experiences of increase case finding of child TB cases, improve treatment outcomes, and the successful implementation of contact screening and management by strengthening of child TB services at peripheral health facilities [2,30]. IMCI should be involved to find and refer from community to child TB sites. In order to improve geographic accessibility, we think there needs to be improvements in two areas. First, to improve identification of children who may have TB at PHC level and need closer examination. Secondly, to improve referral pathways for children with TB from community.

A strength of this study is that it covers almost all of Pakistan, measuring geographical access to health services in a way not done before in Pakistan. A limitation of this study was that we did not have any data on health care in private sector, which is very big in Pakistan. But only <5% of these private facilities are given roles in the national TB control program with diagnosis and management, even though many treat their patients not following the national guidelines [31]. Also, data on settlements and health facilities used in this study were extracted from the Humanitarian Data Exchange website with numbers from 2018, and some changes may have occurred since then. We did not have individual data for geographical access indicators, such as distance, population living within 5 km of health facilities, in order to do cross-sectional analysis, but we could analyse by groups in an ecological study. Finally, we did not have data for the population of provinces of GB (0.5%) and AJK (2%). This study is secondary analysis of different existing data sources; validity of data cannot be assured.

## Conclusion

There was high geographical accessibility to general primary health services in Pakistan, while geographical access to specialised child TB is lower with consequent longer distance to care. Geographical accessibility can be improved by integrated IMCI approach involving Lady Health Workers, and creating a closer link to higher level to improve referral system particularly for distant communities.

## Paper context

Geographical access to child TB health care has direct impact on burden of TB. In Pakistan, child TB services are mainly limited to secondary/tertiary care level. We aimed to determine the geographical access to child TB services in Pakistan. This study reveals low coverage of specialist care for childhood TB with consequent longer distance to care. Integrated IMCI approach involving Lady Health Workers and improved referral system could improve geographical accessibility of child TB care.

## References

[1] UNOPS. Every day more than 650 children die from TB. 2019

[2] UNICEF. Strengthening community and primary health systems for tuberculosis. A consultation on childhood TB integration. 2016.

[3] World Health Organization. Roadmap for childhood tuberculosis towards zero deaths. Geneva(Switzerland): WHO; 2013.

[4] World Health Organization. Global Tuberculosis Report 2021. Geneva, Switzerland:WHO; 2021.

[5] World Health Organization. Roadmap towards ending TB in children and adolescents. Geneva Switzerland: WHO; 2018.

[6] Fluegge K, Malone LL, Nsereko M, et al. Impact of geographic distance on appraisal delay for active TB treatment seeking in Uganda: a network analysis of the Kawempe community health cohort study. BMC Public Health. 2018 Jun 26;18:1–8. 10.1186/s12889-018-5648-6 PMC6019214 29940918

[7] Robsky KO, Robsky KO, Hughes S, et al. Is distance associated with tuberculosis treatment outcomes? A retrospective cohort study in Kampala, Uganda. BMC Infect Dis. 2020 Jun 11;20:1–9. 10.1186/s12879-020-05099-z PMC7291553 32527306

[8] Tadesse T, Demissie M, Berhane Y, et al. Long distance travelling and financial burdens discourage tuberculosis DOTs treatment initiation and compliance in Ethiopia: a qualitative study. BMC Public Health. 2013;13:424. 23634650 10.1186/1471-2458-13-424 PMC3644232

[9] Storla DG, Yimer S, Bjune GA. A systematic review of delay in the diagnosis and treatment of tuberculosis. BMC Public Health. 2008 Jan 14;8:1–9. cited 2021 Dec 28. 18194573 10.1186/1471-2458-8-15 PMC2265684

[10] van Gurp M, Rood E, Fatima R, et al. Finding gaps in TB notifications: spatial analysis of geographical patterns of TB notifications, associations with TB program efforts and social determinants of TB risk in Bangladesh, Nepal and Pakistan. BMC Infect Dis. 2020 Dec 10;20:490. 32650738 10.1186/s12879-020-05207-z PMC7350590

[11] Fatima R, Haq MU, Yaqoob A, et al. Delivering patient-centered care in a fragile state: using patient-pathway analysis to understand tuberculosis-related care seeking in Pakistan. J Infect Dis. 2017;216:S733–9. 29117348 10.1093/infdis/jix380 PMC5853661

[12] Ahmed SAKS, Ajisola M, and Azeem K, et al. Impact of the societal response to COVID-19 on access to healthcare for non-COVID-19 health issues in slum communities of Bangladesh, Kenya, Nigeria and Pakistan: results of pre-COVID and COVID-19 lockdown stakeholder engagements. BMJ Glob Heal. 2020 Aug 1;5(8): 1–17. 10.1136/bmjgh-2020-003042 PMC7443197 32819917

[13] Legido-Quigley H, Naheed A, Asita De Silva H, et al. Patients’ experiences on accessing health care services for management of hypertension in rural Bangladesh, Pakistan and Sri Lanka: a qualitative study. PLoS One. 2019 Jan 1;14:e0211100. 30682093 10.1371/journal.pone.0211100 PMC6347162

[14] Mcnojia SZ, Saleem S, Feroz A, et al. Exploring women and traditional birth attendants’ perceptions and experiences of stillbirths in district Thatta, Sindh, Pakistan: a qualitative study. Reprod Health. 2020 Jan 13;17:1–11. 31931824 10.1186/s12978-020-0852-0 PMC6958748

[15] Pakistan Bureau of Statistics. 6th Population and housing census 2017. Islamabad(Pakistan): Government of Pakistan; 2017.

[16] Pakistan bureau of statistics. National health accounts Pakistan 2013-14. Islamabad(Pakistan): Government of Pakistan; 2015.

[17] Home. LandScan^TM^. (cited 2021 Dec 11). Available from: https://landscan.ornl.gov/

[18] World Health Organization. 100 core health indicators. Geneva(Switzerland): WHO; 2015.

[19] World Health Organization. Global reference list of 100 core health indicators (plus health-related SDGs). Geneva(Switzerland): WHO; 2018.

[20] National TB Control Program. Revised: doctor’s desk guide management of childhood tuberculosis. Islamabad(Pakistan): Ministry of Health; 2017.

[21] National TB Control Program. The Pakistan TB joint program review mission February 11-23, 2019. Islamabad(Pakistan): Ministry of Health; 2019.

[22] Ashiagbor G, Ofori-Asenso R, Forkuo EK, et al. Measures of geographic accessibility to health care in the Ashanti region of Ghana. Sci African. 2020 Sep 1;9:e00453.

[23] Fatima R, Yaqoob A, Qadeer E, et al. Measuring and addressing the childhood tuberculosis reporting gaps in Pakistan: the first ever national inventory study among children. PLoS One. 2019;14:e0227186. 31887208 10.1371/journal.pone.0227186 PMC6936771

[24] Bedard BA, Younge M, Pettit PA, et al. Using telemedicine for tuberculosis care management: a three county inter-municipal approach. J Med Syst. 2018 Jan 1; 42. DOI:10.1007/s10916-017-0872-7. 29181590

[25] Huang GKL, Pawape G, and Taune M, et al. Telemedicine in resource-limited settings to optimize care for multidrug-resistant tuberculosis. Front Public Health. 2019;7(222):1–5. 31457000 10.3389/fpubh.2019.00222 PMC6700224

[26] UNICEF & World Health Organization. Management of sick children by community health workers: intervention models and programme examples. 2006.

[27] The Union. A framework for integrating childhood tuberculosis into community-based child health care. 2013.

[28] Hafeez A, Mohamud BK, Shiekh MR, et al. Lady health workers programme in Pakistan: challenges, achievements and the way forward. J Pak Med Assoc. 2011;61:210–215. 21465929

[29] Bechange S, Schmidt E, Ruddock A, et al. Understanding the role of lady health workers in improving access to eye health services in rural Pakistan – findings from a qualitative study. Arch Public Heal. 2021 Dec 1;79:1–12. 10.1186/s13690-021-00541-3 PMC7890803 33597017

[30] Zawedde-Muyanja S, Nakanwagi A, Dongo JP, et al. Decentralisation of child tuberculosis services increases case finding and uptake of preventive therapy in Uganda. Int J Tuberc Lung Dis. 2018 Nov 1;22:1314–1321. 30355411 10.5588/ijtld.18.0025 PMC7237826

[31] National TB Control Program. National Strategic Plan 2020–2023. Islamabad(Pakistan): Government of Pakistan; 2020.

